# The effect of dapagliflozin on cardiorespiratory fitness in patients with coronary heart disease and type 2 diabetes mellitus following PCI: a single-center prospective randomized controlled study

**DOI:** 10.1186/s12872-026-05757-1

**Published:** 2026-03-25

**Authors:** Jiali Yang, Xinru Li, Liangqiu Tang, Wenmao Fan, Aihua Li, Wanming Zhou, Jungang Pang, Qiuxiao Yuan, Ming Zhong, Jinhui Hou, Lan Wang, Wenjiao Liao, Xiangyang Liu

**Affiliations:** 1 The First School of Clinical Medicine, Guangdong Medical University, Zhanjiang, Guangdong 524023 China; 2 Department of Cardiology, Yuebei People’s Hospital Affiliated to Shantou University Medical College, Shaoguan, Guangdong 512026 China

**Keywords:** Dapagliflozin, Cardiorespiratory fitness, Coronary heart disease, Type 2 diabetes mellitus, Flow-mediated dilation, Adverse cardiovascular events

## Abstract

**Objective:**

To evaluate the effect of dapagliflozin on cardiorespiratory fitness (CRF) in patients with coronary heart disease (CHD) and type 2 diabetes mellitus (T2DM) following percutaneous coronary intervention (PCI).

**Methods:**

A total of 180 patients with CHD and T2DM who underwent PCI at Yuebei People’s Hospital between April 2021 and April 2023 were enrolled and randomly divided into the dapagliflozin group and the non-dapagliflozin group. CRF, brachial artery flow-mediated dilation (FMD), as well as the incidence of cardiovascular events and adverse reactions were compared between the two groups.

**Results:**

After 3 months of intervention, compared with the non-dapagliflozin group, the dapagliflozin group exhibited significantly higher peak oxygen uptake (VO_2_peak) (Adjusted mean difference [AMD] in VO_2_peak for the dapagliflozin group: 21.87 ml/kg/min, 95% confidence interval [*CI*]: 21.05–22.69; AMD in VO_2_peak for the non-dapagliflozin group: 19.65 ml/kg/min, 95% *CI*: 18.2–20.48; *P <* 0.001), and a similar beneficial effect was observed for FMD (AMD in FMD for the dapagliflozin group: 5.63%, 95% *CI*: 5.12–6.14; AMD in FMD for the non-dapagliflozin group: 3.22%, 95% *CI*: 2.7–3.74; *P <* 0.001). No statistically significant between-group differences were detected in the remaining indicators (all *P* > 0.05).

**Conclusion:**

Over a 3-month observational period, dapagliflozin improved CRF, vascular endothelial function, and reduced the incidence of adverse cardiovascular events in patients with CHD and T2DM following PCI.

**Trial registration:**

This trial was registered with the Chinese Clinical Trial Registry (ChiCTR), registration number ChiCTR2100047856, on June 27, 2021 (retrospective registration). The study protocol adheres to the CONSORT guidelines.

## Background

Due to changes in lifestyle, dietary patterns, and stress, the prevalence of coronary heart disease (CHD) in China increased from 1990 to 2019. In addition, factors contributing to the rising prevalence of type 2 diabetes mellitus (T2DM) in China include urbanization, population aging, overweight, and obesity. A large-scale national survey in China showed that the prevalence of diabetes among adults was 11.2% (in accordance with the World Health Organization criteria). Diabetes is a significant risk factor for CHD, and the number of patients with comorbidities has been increasing year by year [1–3]. Percutaneous coronary intervention (PCI) is a key component of CHD management, as it maintains vascular patency and prevents recurrent ischemic events. Patients with CHD and T2DM require long-term standardized drug therapy, including antiplatelet agents, antianginal drugs, myocardial oxygen consumption-lowering agents, anti-myocardial remodeling agents, lipid-lowering/anti-inflammatory agents, and hypoglycemic agents [4–8].

However, studies have shown that patients with CHD experience a decline in cardiorespiratory function and impaired exercise tolerance following PCI, particularly those with T2DM [9–11]. The pathophysiological mechanisms underlying this phenomenon are complex and may be associated with the aforementioned factors [12–15]: PCI may induce transient coronary artery spasm and cause endothelial cell injury. Additionally, potential postoperative events such as stent restenosis or thrombosis may persistently impair effective myocardial perfusion. Reduced physical activity in patients following PCI may lead to cardiorespiratory deconditioning, muscle atrophy, and weakness, subsequently resulting in the deterioration of metabolic function, cardiopulmonary function, and overall health status. Patients with T2DM exhibit insulin resistance, advanced glycation end product accumulation, and chronic low-grade inflammation. These factors exacerbate the loss of skeletal muscle mass and function, impair metabolic efficiency, and reduce oxygen utilization capacity.

Cardiorespiratory fitness (CRF) can reflect individual exercise endurance and can be accurately quantitatively determined by cardiopulmonary exercise testing (CPET). CRF is associated with improved survival and decreased incidence of CHD and other comorbidities including hypertension, diabetes, heart failure, atrial fibrillation and stroke. CRF in CHD and obesity patients is lower than in normal individuals [16, 17]. Therefore, improving exercise capacity and reducing the incidence of major adverse cardiovascular events (MACE) in CHD patients with T2DM following PCI has significant academic research value [18–20].

Studies have shown that diabetic patients exhibit higher sodium-glucose cotransporter 2 (SGLT2) mRNA levels and a more rapid upregulation of SGLT2 in cardiomyocytes compared with non-diabetic individuals [21, 22]. SGLT2 inhibitors represent a novel class of hypoglycemic agents, and multiple studies have confirmed their clinical benefits in patients with T2DM [23–25]. Recent research has demonstrated that SGLT2 inhibitors (e.g., dapagliflozin) confer significant cardiovascular benefits in patients with T2DM. They improve both microvascular and macrovascular endothelial function in these patients [26–30]. SGLT2 inhibitors can improve myocardial energy metabolism, promote reverse remodeling of the cardiac structure, potentially enhance pumping function, and ameliorate cardiac diastolic and filling capacities, thereby increasing patients’ exercise tolerance and quality of life [31]. Additionally, SGLT2 inhibitors possess anti-inflammatory and antioxidative properties, improving glucose homeostasis, reducing systemic inflammation, and exerting inhibitory effects on atherosclerotic plaque inflammation and lipid deposition [32–35]. SGLT2 inhibitors can downregulate the excessive expression of transcription factor Jun-D/peroxisome proliferator-activated receptor γ (PPAR-γ), decrease lipid synthesis, promote lipid oxidation, and thereby exert cardioprotective effects. They can alleviate oxidative stress to improve endothelial function and myocardial cell function in patients with heart failure, as well as reduce body fat percentage, increase muscle mass (either relatively or absolutely), and enhance potential exercise capacity [36–40].

Therefore, we speculate that when administered as an adjunctive therapy in patients with CHD and T2DM following PCI, dapagliflozin may increase VO_2_peak, enhance CRF, improve endothelial function, reduce cardiovascular events, and ameliorate quality of life.

## Methods

### Patients and protocols

#### Study population

This is a single-center, prospective, randomized controlled study, with 180 patients enrolled as the research subjects.

##### Inclusion criteria

(1) Patients diagnosed with both CHD and T2DM who had undergone PCI in the Cardiovascular Department of Yuebei People’s Hospital between April 2021 and April 2023; (2) Patients aged 18 and 80 years;

##### Exclusion criteria

(1) Patients allergic to any component of dapagliflozin; (2) Patients with heart failure classified as NYHA (or Killip) Class III–IV; (3) Patients with bronchial asthma; (4) Patients with interstitial lung disease; (5) Patients unable to cooperate with or refusing to undergo CPET;

##### Withdrawal criteria

(1) Patients unable to continue taking dapagliflozin; (2) Patients who failed to complete the three-month follow-up examination;

This study has been approved by the Ethics Committee of Yuebei People’s Hospital Affiliated to Shantou University Medical College.

#### Sample size calculation

The sample size was estimated using the *sample size estimation for comparing two independent sample means in a completely randomized design* method via PASS11 software, with the settings of α = 0.05 and power (1 − β) = 0.8. Relevant literature indicates elderly patients who underwent cardiac surgery and participated in a 4-week early rehabilitation exercise program exhibited a 1.7 ml/kg/min higher VO_2_peak compared with the control group (*P* < 0.05, with an SD of 3.1 ml/kg/min in the exercise group). In conclusion, the sample size was estimated based on the VO_2_peak criteria: a mean increase of 1.7 ml/kg/min and a standard deviation of 3.1 ml/kg/min, resulting in a required sample size of 54 patients per group. Considering a 10% dropout rate, the planned enrollment was 60 patients per group. However, due to the high dropout rate attributable to the COVID-19 pandemic, a total of 180 patients were finally enrolled in the study.

#### Data collection

The collected baseline clinical data included the following: name, gender, age, body mass index (BMI), hypertension history, smoking history, medication history, lipid profile [total cholesterol (TC), triglycerides (TG), high-density lipoprotein cholesterol (HDL-C), low-density lipoprotein cholesterol (LDL-C)], uric acid (UA), fasting plasma glucose (FPG), hemoglobin A1c (HbA_1_c), and brachial artery FMD. CPET was performed to measure multiple parameters, including VO_2_peak. Patients were randomly assigned to two groups: the dapagliflozin group (10 mg/day plus conventional therapies, including antiplatelet agents, antianginal drugs, myocardial oxygen consumption-lowering agents, anti-remodeling agents, lipid-lowering/anti-inflammatory agents, and hypoglycemic agents) and the non-dapagliflozin group (the same conventional therapies without dapagliflozin). The aforementioned indicators were reassessed after 3 months. We observed changes in the primary outcome measure (VO_2_peak) and other outcome measures (FMD). Additionally, adverse cardiovascular events (recurrent angina pectoris, recurrent acute myocardial infarction, and heart failure) and adverse reactions (hypoglycemia, hypotension, fractures, urinary tract infections, etc.) were tracked.

Primarily due to patient loss to follow-up caused by the COVID-19 pandemic, 57 patients in the dapagliflozin group and 56 patients in the non-dapagliflozin group completed the 3-month follow-up and reexamination.

#### CRF assessment

CRF was assessed using a ramp ergometer protocol for symptom-limited CPET, with test termination criteria including a self-rated exertion score of 16–20 and a respiratory exchange ratio (RER) ≥ 1.1. The test was performed using the German JAEGER Master Screen CPX system. The following indicators were obtained: VO_2_peak.

### Statistical analysis

Multiple imputation was used for missing data imputation. The normality assumption was assessed through visual inspection of quantile-quantile (Q-Q) plots and confirmed with the Shapiro-Wilk test. Continuous variables with a normal distribution were expressed as mean ± standard deviation (mean ± SD), while those with a non-normal distribution were presented as median (interquartile range, IQR); categorical variables were expressed as frequencies. Intragroup differences before and after treatment were analyzed using paired t-tests or paired Wilcoxon signed-rank tests. Normally distributed variables intergroup differences were analyzed using the analysis of covariance (ANCOVA), and non-normally distributed variables intergroup differences were analyzed using the Quade’s nonparametric analysis of covariance, in which both the outcome and continuous covariates were converted to ranks. The independent association between dapagliflozin treatment and the risks of adverse cardiovascular events and treatment-related adverse reactions were analyzed using the multivariable logistic regression analyses. All statistical analyses were performed using SPSS software (Version 22.0, IBM Corp, Chicago, IL, USA).

## Results

**Fig. 1:**
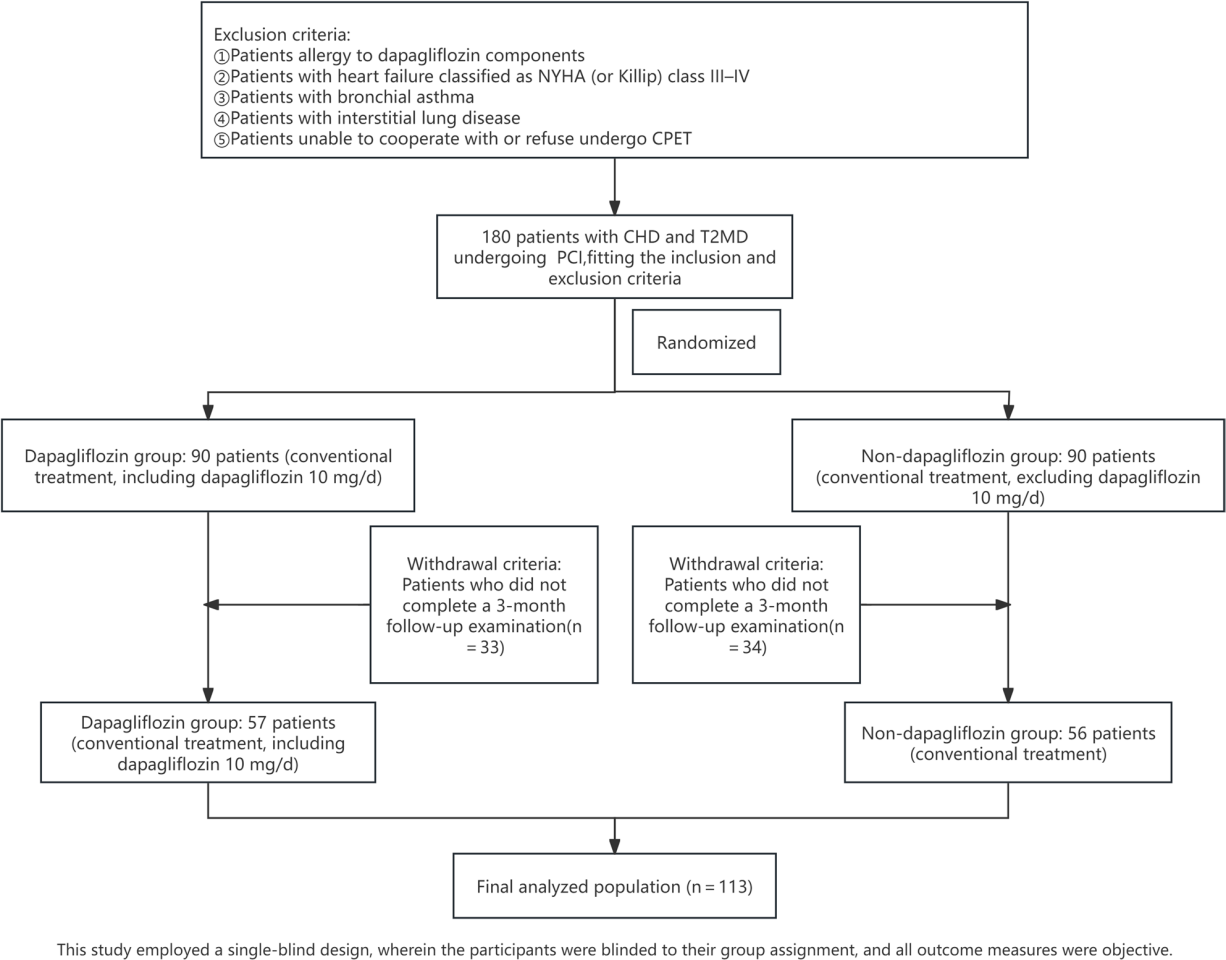
Flowchart

From April 2021 to April 2023, after applying the exclusion criteria, a total of 180 patients diagnosed with CHD and T2DM who had undergone PCI were ultimately included. After 3 months, 33 cases in the dapagliflozin group and 34 cases in the non-dapagliflozin group were lost to reexamination. The final analysis included 57 patients in the dapagliflozin group and 56 patients in the non-dapagliflozin group (see Fig. 1).

### Baseline demographic, procedural, and clinical characteristics

Demographic and procedural characteristics are summarized (see Table 1). Of the 57 patients in the dapagliflozin group, 91.23% were male, with a mean age of 57.63 ± 9.99 years. Of the 56 patients in the non-dapagliflozin group, 73.21% were male, with a mean age of 60.13 ± 10.37 years. Intergroup comparison revealed a statistically significant difference in sex (χ² (1) = 6.293, *P* = 0.012). No significant differences were observed between the two groups in terms of medication categories, age, BMI, and comorbidities.

**Table 1:** Baseline demographic, procedural, and clinical characteristics

Group (n)	The Dapagliflozin Group (57)	The Non-dapagliflozin Group (56)	t/X²	P
Male, n (%)	52(91.23)	41(73.21)	6.293	0.012
Age (year)	57.63±9.99	60.13±10.37	-1.302	0.196
BMI (kg/m2)	25.28±3.86	24.08±3.11	1.814	0.072
Comorbidity, n (%)	-	-	-	-
Smoking	25(43.86）	16(28.57）	2.856	0.091
Hypertension	25(43.86）	32(57.14）	1.994	0.158
Medication, n (%)	-	-	-	-
Aspirin	54(94.70)	50(89.30)	1.145	0.321
Clopidogrel	56(98.24)	54(96.43)	0.361	0.618
Statins	55(94.74)	52(92.86)	0.742	0.438
Cholesterol Absorption Inhibitors	12(21.05)	15(26.79)	0.511	0.514
ACEI	8(14.03)	6(10.71)	0.277	0.776
ARB	25(43.86)	27(48.21)	0.216	0.707
ARNI	11(19.30)	15(26.79)	0.894	0.378
β-blockers	45(78.95)	40(71.43)	0.857	0.390
Nitrates	19(33.33)	27(48.21)	2.592	0.128
Biguanides	22(38.60)	28(50.00)	1.489	0.258
DPP-4 inhibitors	9(15.79)	14(25.00)	1.478	0.250
Sulfonylureas	18(31.58)	24(42.86)	1.539	0.215
Insulin/Insulin Analogs	24(42.11)	30(53.57)	1.735	0.256
MRAs	7(12.28)	6(10.71)	0.108	0.776
VO_2_peak(ml/kg/min)	20.10±3.39	18.47±4.68	2.122	0.036
FMD (%)	2.59±1.65	4±2.06	-4.001	0.000
FPG (mmol/l)	5.93(5.12,7.52)	5,49(4.78,7.58)	0.672	0.504
UA (μmol/l)	372.94±103.55	382.59±97.91	-0.509	0.612
TC (mmol/l)	4.63±1.16	4.97±1.77	-1.182	0.240
TG (mmol/l)	1.21(0.84,2.08)	1.43(0.84,2.09)	-0.850	0.395
HDL-C (mmol/l)	1.13±0.26	1.07±0.24	1.329	0.187
LDL-C (mmol/l)	2.75±1.01	2.88±1.24	-0.585	0.559
HbA_1_c（%）	6.42(5.45,7.94)	5.95(5.40,7.27)	1.002	0.318

- Indicates no test statistic

**Fig. 2:**
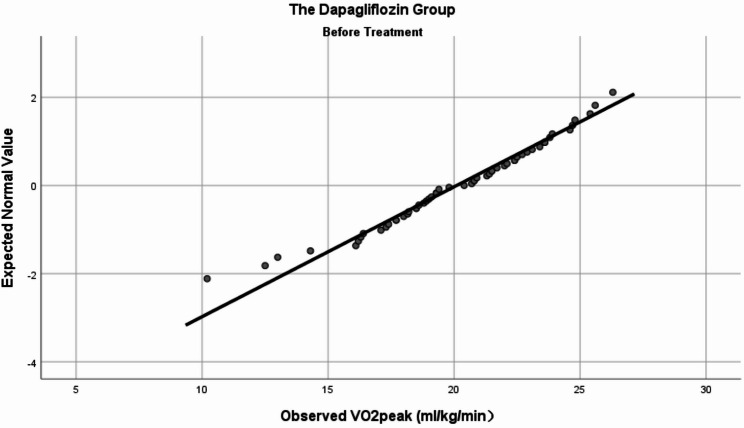
Normal Q-Q plot of baseline VO_2_peak data in the dapagliflozin group

**Fig. 3:**
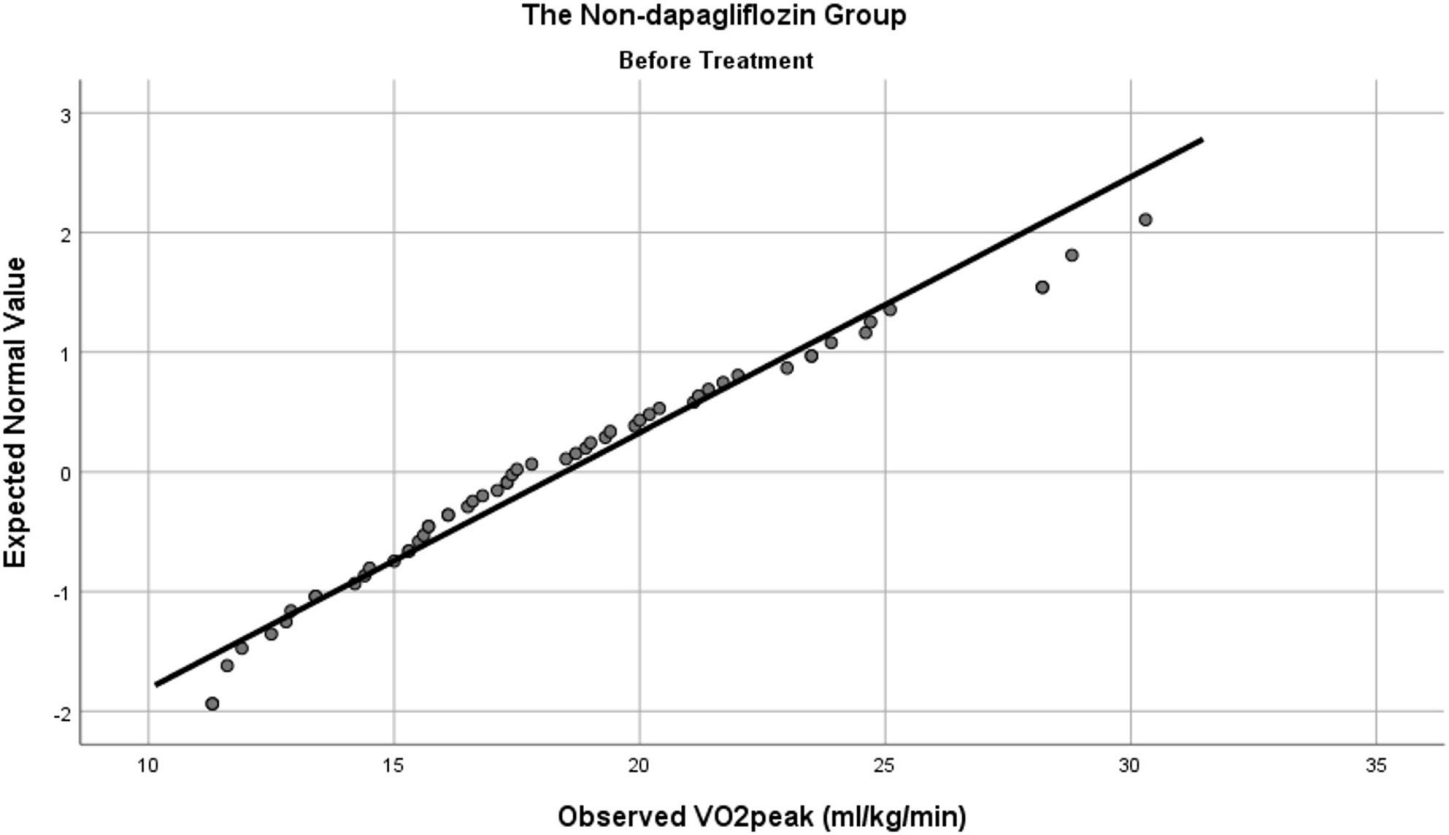
Normal Q-Q plot of baseline VO_2_peak data in the non-dapagliflozin group

Prior to comparative analysis, a normality test was conducted for all baseline variables. VO_2_peak (Figs. 2 and 3), FMD, UA, TC, HDL-C, and LDL-C were normally distributed, whereas the remaining indicators did not. The dapagliflozin group showed a higher VO_2_peak (20.10 ± 3.39 ml/kg/min) compared with the non-dapagliflozin group (18.47 ± 4.68 ml/kg/min) (*t* (111) = 2.122, *P* = 0.036). Conversely, the dapagliflozin group exhibited a lower FMD (2.59 ± 1.65%) than the non-dapagliflozin group (4.00 ± 2.06%) (*t* (111) = -4.001, *P <* 0.001). No statistically significant differences were found in the remaining indicators (including those analyzed with Mann-Whitney *U* tests) (see Table 1).

### Intragroup comparison of observation indicators before and after treatment in the dapagliflozin group and the non-dapagliflozin group

**Fig. 4:**
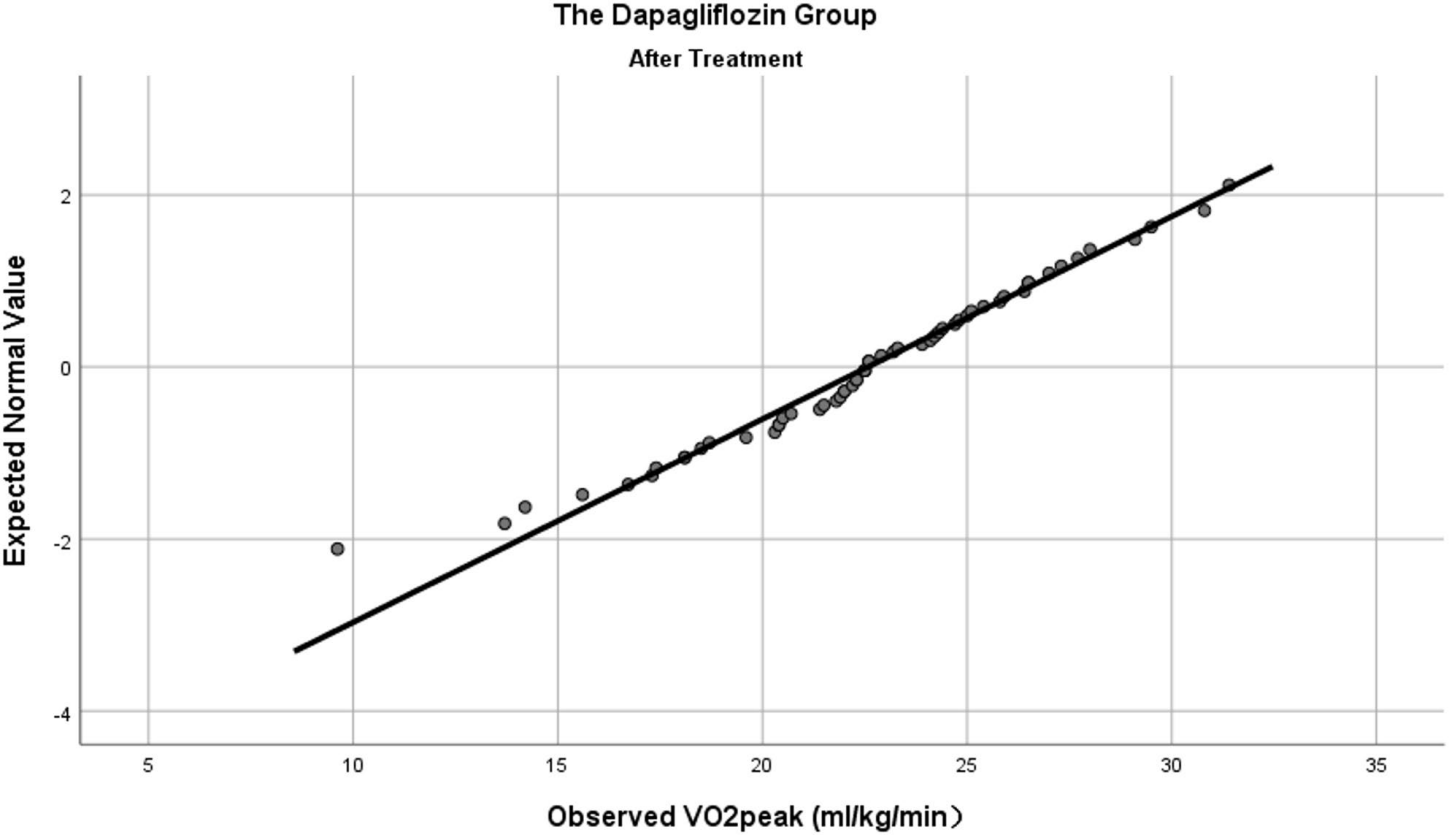
Normal Q-Q plot of VO_2_peak in the dapagliflozin group after treatment

Prior to comparing the observation indicators before and after treatment within the dapagliflozin group, we assessed the normality of all variables. VO_2_peak (Fig. 4), UA, HDL-C, LDL-C, and HbA_1_c were normally distributed, whereas the remaining indicators were not. Statistically significant differences were observed in VO_2_peak (20.10 ± 3.39 vs. 22.58 ± 4.2 ml/kg/min, *P <* 0.001), FMD (2.59 ± 1.65 vs. 5.29 ± 2.21%, *p <* 0.001), TC (4.63 ± 1.16 vs. 4.52 ± 3.5 mmol/L, *P <* 0.001), and LDL-C (2.75 ± 1.01 vs. 2.31 ± 0.9 mmol/L, *P <* 0.001). No statistically significant differences were found in the remaining indicators (see Table 2).

**Table 2:** Comparison of observation indicators before and after treatment in the dapagliflozin group

Group (*n*)	Before treatment (57)	After treatment (57)	Δ (Post-Pre) (57)	t/Z	*P*
VO_2_peak(ml/kg/min)	20.10 ± 3.39	22.58 ± 4.24	2.48 ± 3.12	-5.99	< 0.001
FMD (%)	2.59 ± 1.65	5.29 ± 2.21	2.7 ± 2.14	-6.1	< 0.001
FPG (mmol/l)	5.93(5.12,7.52)	5.71(5.26,6.41)	-0.27(-1.72,0.71)	1.823	0.069
UA (µmol/l)	372.94 ± 103.55	401.03 ± 131.43	16.00(-41.39,63.94)	-1.635	0.108
TC (mmol/l)	4.63 ± 1.16	4.52 ± 3.58	-0.69(-1.41,0.06)	4.796	< 0.001
TG (mmol/l)	1.21(0.84,2.08)	1.43(0.98,2.06)	0.09(-0.56,0.59)	-0.405	0.689
HDL-C (mmol/l)	1.13 ± 0.26	1.1 ± 0.22	-0.03 ± 0.25	0.87	0.388
LDL-C (mmol/l)	2.75 ± 1.01	2.31 ± 0.93	-0.44 ± 1.1	3.002	0.004
HbA_1_c(%)	6.42(5.45,7.94)	6.12 ± 1.45	-0.43 ± 2.33	1.389	0.17

Comparison between before and after treatment

As part of the analysis, the change from baseline (Δ) for each outcome was calculated as the post-treatment value minus the pre-treatment value (Δ = Post – Pre).

**Fig. 5:**
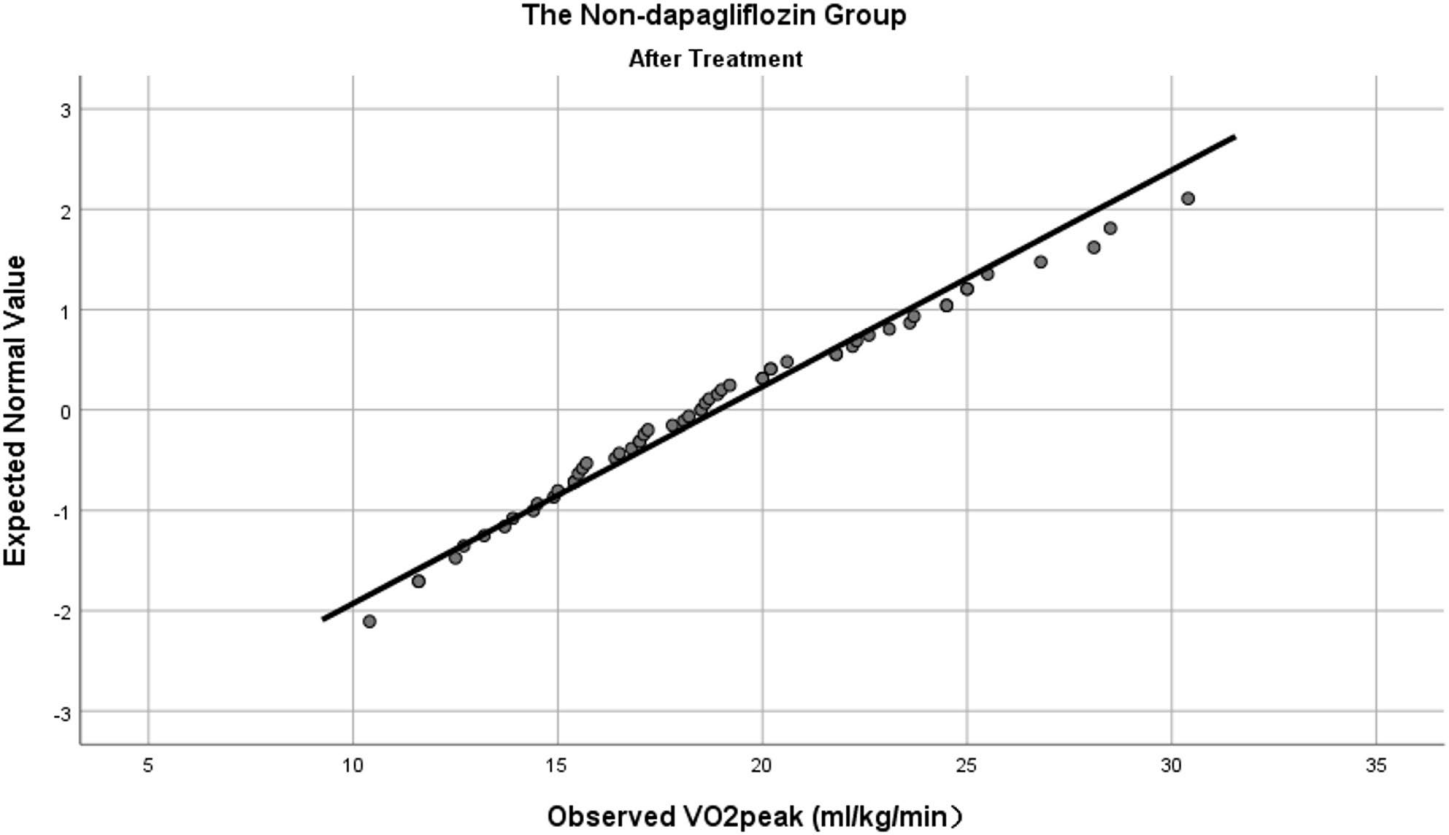
Normal Q-Q plot of VO_2_peak in the non-dapagliflozin group after treatment

Prior to comparing the observation indicators before and after treatment within the non-dapagliflozin group, we assessed the normality of all variables. VO_2_peak (Fig. 5), UA, HDL-C, and LDL-C were normally distributed, whereas the remaining indicators were not. Statistically significant differences were observed in TC (4.97 ± 1.77 vs. 4.16 ± 1.48 mmol/L, *P <* 0.001) and LDL-C (2.88 ± 1.20 vs. 2.17 ± 0.82 mmol/L, *P <* 0.001). No statistically significant differences were found in the remaining indicators (see Table 3).

**Table 3:** Comparison of observation indicators before and after treatment in the non-dapagliflozin group

Group (*n*)	Before treatment (56)	After treatment (56)	Δ (Post-Pre) (56)	t/Z	*P*
VO2peak(ml/kg/min)	18.47 ± 4.68	18.93 ± 4.63	0.46 ± 3.09	-1.107	0.273
FMD (%)	4 ± 2.06	3.56 ± 1.82	-0.43 ± 1.99	1.63	0.109
FPG (mmol/l)	5.49(4.78,7.58)	5.92(5.37,7.37)	-0.60(-1.70,0.70)	-0.29	0.775
UA (µmol/l)	382.59 ± 97.91	395.42 ± 116.26	14.73(-42.50,71.53)	-0.992	0.325
TC (mmol/l)	4.97 ± 1.77	4.16 ± 1.48	-0.84(-1.79,0.07)	3.532	< 0.001
TG (mmol/l)	1.43(0.84,2.09)	1.32(0.96,2.45)	-0.01(-0.51,0.61)	0.139	0.892
HDL-C (mmol/l)	1.07 ± 0.24	1.13 ± 0.28	0.06 ± 0.24	-1.84	0.071
LDL-C (mmol/l)	2.88 ± 1.24	2.17 ± 0.82	-0.71 ± 1.04	5.096	< 0.001
HbA1c(%)	5.95(5.40,7.27)	5.90(5.34,7.59)	0.08 ± 2.48	0.063	0.952

Comparison between before and after treatment

As part of the analysis, the change from baseline (Δ) for each outcome was calculated as the post-treatment value minus the pre-treatment value (Δ = Post – Pre).

### Comparison of observation indicators between the two groups after treatment

To address baseline imbalances in sex, VO_2_peak and FMD, we employed distinct analytical strategies based on data distribution characteristics: normally distributed outcomes were analyzed using analysis of covariance (ANCOVA); outcomes that deviated from normality were analyzed using the Quade’s nonparametric analysis of covariance, in which both the outcome and continuous covariates were converted to ranks. VO_2_peak (Figs. 4 and 5), FMD, UA, TC, HDL-C and LDL-C were normally distributed, whereas the remaining indicators were not.

Compared with the non-dapagliflozin group, the dapagliflozin group exhibited significantly higher VO_2_peak (AMD in VO_2_peak for the dapagliflozin group: 21.87 ml/kg/min, 95% *CI*: 21.05–22.69; AMD in VO_2_peak for the non-dapagliflozin group: 19.65 ml/kg/min, 95% *CI*: 18.2–20.48; *P <* 0.001), and a similar beneficial effect was observed for FMD (AMD in FMD for the dapagliflozin group: 5.63%, 95% *CI*: 5.12–6.14; AMD in FMD for the non-dapagliflozin group: 3.22%, 95% *CI*: 2.7–3.74; *P <* 0.001). No statistically significant between-group differences were detected in the remaining indicators. (see Tables 4 and 5).

**Table 4:** Results of Analysis of Covariance (ANCOVA) for normality observation indicators

Group (*n*)	The Dapagliflozin Group Adjusted Mean (95% CI) (57)	The Non-dapagliflozin Group Adjusted Mean (95% CI) (56)	F(df)	*P*	Partial η² < 0.001
VO_2_peak(ml/kg/min)	21.87(21.05–22.69)	19.65(18.82–20.48)	12.78(1,108)	0.001	0.106
FMD (%)	5.63(5.12–6.14)	3.22(2.70–3.74)	38.96(1,108)	< 0.001	0.265
UA (µmol/l)	399.90(367.21-432.58)	396.57(363.57-429.58)	0.02(1,108)	0.893	< 0.001
TC (mmol/l)	4.46(3.70–5.22)	4.22(3.45–4.99)	0.17(1,108)	0.683	0.002
HDL-C (mmol/l)	1.10(1.04–1.17)	1.12(1.05–1.19)	0.12(1,108)	0.725	0.001
LDL-C (mmol/l)	2.32(2.07–2.56)	2.17(1.93–2.42)	0.62(1,108)	0.432	0.006

For normally distributed outcomes (as indicated above), analysis of covariance (ANCOVA) was employed. Results are presented as adjusted mean differences with 95% confidence intervals (CIs) for the comparison between the dapagliflozin group and the non-dapagliflozin group

**Table 5:** Results of nonparametric analysis of covariance (Quade’s ANCOVA) for non-normality observation indicators

Group (n)	The Dapagliflozin Group Median (IQR) (57)	The Non-dapagliflozin Group Median (IQR) (56)	F(df)	P	Partial η²
FPG (mmol/l)	5.71(5.26-6.41)	5.92(5.37-7.37)	0.004(1,108)	0.947	< 0.001
TG (mmol/l)	1.43(0.98-2.06)	1.32(0.96-2.45)	0.03(1,108)	0.864	< 0.001
HbA1c（%）	5.80(5.26-7.05)	5.90(5.34-7.59)	2.50(1,108)	0.117	0.023

For non-normally distributed outcomes (as indicated above), Quade's nonparametric analysis of covariance was applied. Results are presented as F(df) and P value for the comparison between the dapagliflozin group and the non-dapagliflozin group

### Comparison of the incidence of adverse cardiovascular events and adverse reactions between the dapagliflozin group and the non-dapagliflozin group

To determine the independent association between dapagliflozin treatment and the risks of adverse cardiovascular events and treatment-related adverse reactions, we performed multivariable logistic regression analyses adjusted for sex, baseline VO_2_peak, and baseline FMD. Compared with the non-dapagliflozin group, the dapagliflozin group had a significantly lower incidence of recurrent angina pectoris (adjusted odds ratio [aOR] = 0.125, 95% *CI*: 0.016–0.995 P = 0.049), heart failure (aOR = 0.155, 95% *CI*: 0.025–0.953, P = 0.044), and total adverse cardiovascular events (aOR = 0.105, 95% *CI*: 0.026–0.419, P = 0.001). No statistically significant between-group differences were observed in the incidence of acute myocardial infarction or hypoglycemia (the only adverse reaction analyzed in the present study) (all *P* > 0.05). (see Table 6). Hypoglycemia was the only adverse reaction observed in this study. No adverse events such as hypotension, fractures, urinary tract infections were observed in either group.

**Table 6:** Comparison of the incidence of adverse cardiovascular events and adverse reactions at 3-month follow-up

Incidence	Recurrent angina pectoris	Acute myocardial infarction	Heart failure	Total number of adverse cardiovascular events	Adverse reactions (hypo-glycaemia)
Dapagliflozin group(n/%)	3(5.26)	0(0)	2(3.51)	5(8.77)	1(1.76)
Non-dapagliflozin group(n/%)	4(7.14)	1(1.79)	8(14.29)	13(23.21)	1(1.79)
Adjusted OR < 0.001	0.125	< 0.001	0.155	0.105	1.50
95% CI	0.016-0.995	0.00-	0.025-0.953	0.026-0.419	0.05-45.46
P	0.049	0.997	0.044	0.001	0.816

## Discussion

The prevalence of T2DM is increasing annually, imposing a substantial global economic burden, particularly in developing countries. CHD is a common complication in patients with T2DM, characterized by endothelial dysfunction and microcirculatory hemodynamic alterations, which results in high morbidity and disability rates [41–45]. Dapagliflozin, a SGLT2 inhibitor, exerts beneficial effects on both T2DM and cardiovascular diseases. It alleviates cardiac microvascular injury and endothelial dysfunction via the XO-SERCA2-CaMKII-cofilin pathway during ischemia-reperfusion injury (IRI). Additionally, by activating the protein kinase B (AKT/PKB) pathway and other signaling cascades, it attenuates cardiac hypertrophy, inflammation, and cellular stress, thereby improving cardiac and endothelial function and ameliorating metabolic dysregulation. Collectively, these mechanisms protect cardiac function in patients with T2DM [46, 47]. A robust body of evidence has established that an elevation in VO_2_peak is significantly associated with improved cardiovascular outcomes. A meta-analysis demonstrated that each 1 ml/kg/min increment in VO_2_peak correlates with a 7%–17% reduction in the risk of all-cause mortality [48]. In a study focusing exclusively on patients with CHD, every 1 ml/kg/min increase in VO_2_peak was linked to an approximate 9% relative reduction in all-cause mortality risk [49].

A decrease in VO_2_peak is observed in patients with CHD and T2DM. PCI can resolve coronary artery stenosis but does not significantly improve VO_2_peak [8, 50, 51]. Despite anatomical correction, its effect on VO_2_peak remains limited [6, 52]. The results of this study demonstrated a statistically significant intragroup difference in VO_2_peak before and after treatment in the dapagliflozin group (*P* < 0.001), with a mean increase in VO_2_peak of 2.48 ml/kg/min. After controlling for baseline imbalances in sex, VO_2_peak, and FMD, intergroup differences in VO_2_peak before and after treatment in the dapagliflozin group were statistically significant ([AMD] in VO_2_peak for the dapagliflozin group: 21.87 ml/kg/min, 95% confidence interval [CI]: 21.05–22.69; AMD in VO_2_peak for the non-dapagliflozin group: 19.65 ml/kg/min, 95% CI: 18.20–20.48; *P* < 0.001). These findings suggest that dapagliflozin can further improve VO_2_peak in patients with CHD and T2DM who have undergone PCI (*P* < 0.001).

Vascular endothelial damage and dysfunction are early signs of atherosclerosis. They act as sentinels of cardiovascular health and are key prognostic indicators for future cardiovascular events [53]. This study revealed significant intragroup differences in FMD before and after treatment in the dapagliflozin group were statistically significant (*p* < 0.001). After controlling for baseline imbalances in sex, VO_2_peak, and FMD, intergroup differences in FMD before and after treatment in the dapagliflozin group were statistically significant (AMD in FMD for the dapagliflozin group: 5.63%, 95% *CI*: 5.12–6.14; AMD in FMD for the non-dapagliflozin group: 3.22%, 95% *CI*: 2.7–3.74; *P <* 0.001). This finding suggests that dapagliflozin can further improve FMD in patients with CHD and T2DM who have undergone PCI (*P* < 0.001).

Accumulating evidence has established that sodium-glucose cotransporter 2 (SGLT2) inhibitors, including dapagliflozin, exert substantial cardiovascular protective effects in patients with T2DM [26–30]. Consistent with these findings, our study demonstrated that dapagliflozin treatment was associated with a significantly reduced risk of adverse cardiovascular events in patients with CHD complicated by T2DM who had undergone PCI. After 3 months of follow-up and adjustment for baseline imbalances in sex, VO_2_peak, and FMD, the dapagliflozin group exhibited a notably lower incidence of recurrent angina pectoris (5.26% vs. 7.14% in the non-dapagliflozin group; aOR = 0.125, 95% CI: 0.016–0.995 P = 0.049) and heart failure (3.51% vs. 14.29% in the non-dapagliflozin group; aOR = 0.155, 95% CI: 0.025–0.953, P = 0.044). Moreover, the overall incidence of total adverse cardiovascular events was significantly lower in the dapagliflozin group compared with the non-dapagliflozin group (8.77% vs. 23.21%; aOR = 0.105, 95% CI: 0.026–0.419, P = 0.001). Notably, no statistically significant between-group differences were observed in the incidence of acute myocardial infarction or treatment-related adverse reactions. It is worth mentioning that hypoglycemia was the only adverse reaction documented in the present study; no cases of hypotension, fractures, urinary tract infections, or renal dysfunction were reported in either group (all *P* > 0.05), which further supports the favorable safety profile of dapagliflozin in this specific patient population.

In summary, a 3-month follow-up data analysis yielded significant findings, confirming that for patients with CHD and T2DM who underwent PCI, the addition of dapagliflozin to secondary prevention therapy confers notable short-term benefits, with a significant improvement in VO₂peak and FMD observed during this period. Furthermore, dapagliflozin effectively reduces the incidence of adverse cardiovascular events (e.g., recurrent angina pectoris, heart failure) in this patient population without increasing the risk of treatment-related adverse reactions, thereby providing cardiovascular benefits that extend beyond glycemic control.

### Study limitations

However, this study has several limitations. First, although the initial sample size was adequately calculated with allowance for potential dropouts, the actual dropout rate attributable to the COVID-19 pandemic exceeded the anticipated level. While the study was ultimately completed by expanding the enrollment scale, and the robustness of the primary conclusions has been verified via sensitivity analysis, the high dropout rate may have introduced selection bias, thereby compromising the generalizability of the study results. Second, post-randomization, imbalances were observed between the two groups in terms of gender, baseline VO_2_peak, and FMD. Despite statistical adjustments for these factors, residual confounding bias may still persist, which may have exerted a certain influence on the estimated effect size and the reliability of the conclusions. Third, this study employed a single-center design with a 3-month follow-up duration, primarily reflecting the short-term effects of the drug; its medium- to long-term cardioprotective efficacy and safety thus remain to be confirmed through extended follow-up. Fourth, although the outcome assessors involved in evaluating efficacy and adverse events were blinded, which helped reduce measurement bias, the relatively small sample size resulted in a low number of observed adverse events (e.g., hypotension, fractures, urinary tract infections). This limited event count restricts our ability to fully characterize the drug’s safety profile, particularly regarding the detection of rare but clinically important adverse reactions. Furthermore, the subjective components inherent in the clinical judgment and adjudication criteria for adverse events may still introduce some degree of assessment bias, which should be acknowledged when interpreting the safety results. Fifth, although improvements in short-term clinical endpoints were observed in this study, the long-term changes in key functional indicators such as cardiopulmonary exercise capacity and FMD were not continuously monitored or analyzed. Therefore, the long-term trajectory of treatment benefits could not be fully characterized. Finally, the specific molecular and physiological mechanisms underlying the beneficial effects of dapagliflozin (e.g., relative contributions to myocardial metabolism, systemic inflammation, or vascular function) remain elusive and warrant further elucidation through mechanistic investigations in the future.

Based on the findings and limitations of this study, future research directions could include: (1) Conducting multicenter, large-sample randomized controlled trials to validate the generalizability of the conclusions, mitigate selection bias from high dropout rates, enhance the detection of adverse events (especially rare ones) related to small sample sizes, and minimize baseline imbalances between groups; (2) Designing extended follow-up periods (≥ 1 year) to evaluate long-term risk-benefit profiles, confirm dapagliflozin’s medium- to long-term cardioprotective efficacy and safety, monitor long-term changes in key functional indicators (e.g., VO2peak, FMD), and fully characterize the long-term trajectory of treatment benefits; and (3) Leveraging technical modalities such as cardiac imaging (e.g., cardiac magnetic resonance [CMR]) and serum biomarker omics to comprehensively elucidate the mechanisms by which dapagliflozin enhances cardiopulmonary function and improves prognosis in patients with CHD complicated by T2DM.

## Conclusion

In conclusion, the present study demonstrates that dapagliflozin confers significant early benefits by enhancing VO_2_peak, FMD, and reducing the incidence of cardiovascular adverse events in patients with CHD and T2DM who have undergone PCI. These findings highlight the potential of dapagliflozin as an effective therapeutic option for improving cardiopulmonary fitness and cardiovascular outcomes in this high-risk patient group, providing valuable empirical support for its clinical use in the post-PCI setting.

## Data Availability

The datasets generated and/or analyzed during the current study are not publicly available due to them containing information that could compromise the privacy of patients but are available from the corresponding author on reasonable request.

## Abbreviations

CRF: Cardiorespiratory fitness
CHD: Coronary heart disease
T2DM: Type 2 diabetes mellitus
PCI: Percutaneous coronary intervention
FMD: Flow-mediated dilation
VO2peak: Peak oxygen uptake
AMD: Adjusted mean difference
CI: Confidence interval
CPET: Cardiopulmonary exercise testing
MACE: Major adverse cardiovascular events
SGLT2: Sodium-glucose cotransporter 2
PPAR-γ: Peroxisome proliferator-activated receptor γ
BMI: Body mass index
TC: Total cholesterol
TG: Triglycerides
HDL-C: High-density lipoprotein cholesterol
LDL-C: Low-density lipoprotein cholesterol
UA: Uric acid
FPG: Fasting plasma glucose
HbA1c: Hemoglobin A1c
Q-Q: Quantile-quantile
aOR: Adjusted odds ratio
CMR: Cardiac magnetic resonance
IRI: Ischemia-reperfusion injury
AKT/PKB: Protein kinase B
