# Correction: The effect of dapagliflozin on cardiorespiratory fitness in patients with coronary heart disease and type 2 diabetes mellitus following PCI: a single-center prospective randomized controlled study

**DOI:** 10.1186/s12872-026-06036-9

**Published:** 2026-06-02

**Authors:** Jiali Yang, Xinru Li, Liangqiu Tang, Wenmao Fan, Aihua Li, Wanming Zhou, Jungang Pang, Qiuxiao Yuan, Ming Zhong, Jinhui Hou, Lan Wang, Wenjiao Liao, Xiangyang Liu, BMC Cardiovascular

**Affiliations:** 1 The First School of Clinical Medicine, Guangdong Medical University, Zhanjiang, Guangdong 524023 China; 2 Department of Cardiology, Yuebei People’s Hospital, Shantou University Medical College, Shaoguan, Guangdong 512026 China

**Correction: BMC Cardiovascular Disorders 26, 386 (2026)**

**https://doi.org/10.1186/s12872-026-05757-1**

Some of the data provided in Table 1 and Table 6 of the article [1] appears to be incorrect.

The following updates should be made to Table 1:

- ACEI medication proportion (Dapagliflozin group): The value “8(10.03)” should be changed to “8(14.03)”.
- Nitrates proportion (Non-dapagliflozin group): The value “38(67.86)” should be changed to “27(48.21)”.
- BMI (kg/m2): The P-value “0.720” contains a decimal error and should be changed to “0.072”.

The corrected version of Table 1 is provided below:

**Table 1:** Baseline demographic, procedural, and clinical characteristics

Group (n)	The Dapagliflozin Group (57)	The Non-dapagliflozin Group (56)	t/X ²	P
Male, n (%)	52(91.23)	41(73.21)	6.293	0.012
Age (year)	57.63±9.99	60.13±10.37	-1.302	0.196
BMI (kg/m2)	25.28±3.86	24.08±3.11	1.814	0.072
Comorbidity, n (%)	-	-	-	-
Smoking	25(43.86）	16(28.57）	2.856	0.091
Hypertension	25(43.86）	32(57.14）	1.994	0.158
Medication, n (%)	-	-	-	-
Aspirin	54(94.70)	50(89.30)	1.145	0.321
Clopidogrel	56(98.24)	54(96.43)	0.361	0.618
Statins	55(94.74)	52(92.86)	0.742	0.438
Cholesterol Absorption Inhibitors	12(21.05)	15(26.79)	0.511	0.514
ACEI	8(14.03)	6(10.71)	0.277	0.776
ARB	25(43.86)	27(48.21)	0.216	0.707
ARNI	11(19.30)	15(26.79)	0.894	0.378
β-blockers	45(78.95)	40(71.43)	0.857	0.390
Nitrates	19(33.33)	27(48.21)	2.592	0.128
Biguanides	22(38.60)	28(50.00)	1.489	0.258
DPP-4 inhibitors	9(15.79)	14(25.00)	1.478	0.250
Sulfonylureas	18(31.58)	24(42.86)	1.539	0.215
Insulin/Insulin Analogs	24(42.11)	30(53.57)	1.735	0.256
MRAs	7(12.28)	6(10.71)	0.108	0.776
VO_2_peak(ml/kg/min)	20.10±3.39	18.47±4.68	2.122	0.036
FMD (%)	2.59±1.65	4±2.06	-4.001	0.000
FPG (mmol/l)	5.93(5.12,7.52)	5,49(4.78,7.58)	0.672	0.504
UA (μmol/l)	372.94±103.55	382.59±97.91	-0.509	0.612
TC (mmol/l)	4.63±1.16	4.97±1.77	-1.182	0.240
TG (mmol/l)	1.21(0.84,2.08)	1.43(0.84,2.09)	-0.850	0.395
HDL-C (mmol/l)	1.13±0.26	1.07±0.24	1.329	0.187
LDL-C (mmol/l)	2.75±1.01	2.88±1.24	-0.585	0.559
HbA_1_c（%）	6.42(5.45,7.94)	5.95(5.40,7.27)	1.002	0.318

_- indicates no test statistic_

Additionally, Table 6 requires corrections as the entries in the rows for Adjusted OR < 0.001, 95% CI, and P were incorrect. The updated table is shown below:

Incorrect Table 6

**Table 6:** Comparison of the incidence of adverse cardiovascular events and adverse reactions at 3-month follow-up

Incidence	Recurrent angina pectoris	Acute myocardial infarction	Heart failure	Total number of adverse cardiovascular events	Adverse reactions (hypo-glycaemia)
Dapagliflozin group(n/%)	3(5.26)	0(0)	2(3.51)	5(8.77)	1(1.76)
Non-dapagliflozin group(n/%)	4(7.14)	1(1.79)	8(14.29)	13(23.21)	1(1.79)
Adjusted OR	0.125	0	0.155	0.105	1.50
95% CI	0.016-0.995	0.00-	0.025-0.953	0.026-0.419	0.05-45.46
P	0.049	0.997	0.044	0.001	0.816

**Table 6:** Comparison of the incidence of adverse cardiovascular events and adverse reactions at 3-month follow-up

Incidence	Recurrent angina pectoris	Acute myocardial infarction	Heart failure	Total number of adverse cardiovascular events	Adverse reactions (hypo-glycaemia)
Dapagliflozin group(n/%)	3(5.26)	0(0)	2(3.51)	5(8.77)	1(1.76)
Non-dapagliflozin group(n/%)	4(7.14)	1(1.79)	8(14.29)	13(23.21)	1(1.79)
Adjusted OR	0.089	0	0.096	0.14	0.667
95% CI	0.01-0.85	0.00-	0.01-0.68	0.03-0.58	0.02-20.20
P	0.035	0.997	0.019	0.007	0.816

Corrected Table 6

The original article has been corrected.
